# Response to acute vasodilator challenge and haemodynamic modifications after MitraClip in patients with functional mitral regurgitation and pulmonary hypertension

**DOI:** 10.1093/ehjacc/zuac053

**Published:** 2022-05-07

**Authors:** Alessandro Mandurino-Mirizzi, Andrea Munafò, Claudia Raineri, Giulia Magrini, Romina Frassica, Luca Arzuffi, Laura Scelsi, Annalisa Turco, Marco Ferlini, Fabrizio Gazzoli, Maurizio Ferrario, Stefano Ghio, Luigi Oltrona-Visconti, Gabriele Crimi

**Affiliations:** Division of Cardiology, Fondazione IRCCS Policlinico San Matteo, Piazzale Golgi 1, 27100 Pavia, Italy; University of Pavia, Fondazione IRCCS Policlinico San Matteo, Pavia, Italy; Division of Cardiology, Fondazione IRCCS Policlinico San Matteo, Piazzale Golgi 1, 27100 Pavia, Italy; University of Pavia, Fondazione IRCCS Policlinico San Matteo, Pavia, Italy; Division of Cardiology, Fondazione IRCCS Policlinico San Matteo, Piazzale Golgi 1, 27100 Pavia, Italy; Division of Cardiology, Fondazione IRCCS Policlinico San Matteo, Piazzale Golgi 1, 27100 Pavia, Italy; Division of Cardiology, Fondazione IRCCS Policlinico San Matteo, Piazzale Golgi 1, 27100 Pavia, Italy; Division of Cardiology, Fondazione IRCCS Policlinico San Matteo, Piazzale Golgi 1, 27100 Pavia, Italy; University of Pavia, Fondazione IRCCS Policlinico San Matteo, Pavia, Italy; Division of Cardiology, Fondazione IRCCS Policlinico San Matteo, Piazzale Golgi 1, 27100 Pavia, Italy; Division of Cardiology, Fondazione IRCCS Policlinico San Matteo, Piazzale Golgi 1, 27100 Pavia, Italy; Division of Cardiology, Fondazione IRCCS Policlinico San Matteo, Piazzale Golgi 1, 27100 Pavia, Italy; Division of Cardiology, Fondazione IRCCS Policlinico San Matteo, Piazzale Golgi 1, 27100 Pavia, Italy; Division of Cardiology, Fondazione IRCCS Policlinico San Matteo, Piazzale Golgi 1, 27100 Pavia, Italy; Division of Cardiology, Fondazione IRCCS Policlinico San Matteo, Piazzale Golgi 1, 27100 Pavia, Italy; Division of Cardiology, Fondazione IRCCS Policlinico San Matteo, Piazzale Golgi 1, 27100 Pavia, Italy; Division of Cardiology, Fondazione IRCCS Policlinico San Matteo, Piazzale Golgi 1, 27100 Pavia, Italy; Interventional Cardiology Unit, Cardio-Thoraco Vascular Department (DICATOV), IRCCS Ospedale Policlinico San Martino, Genova, Italy; IRCCS Italian Cardiovascular Network & Department of Internal Medicine, University of Genova, Genova, Italy

**Keywords:** Heart failure, Functional mitral regurgitation, Pulmonary hypertension, MitraClip, Transcatheter mitral valve repair

## Abstract

The effectiveness of transcatheter edge-to-edge repair (TEER) in patients with functional mitral regurgitation (FMR) and pulmonary hypertension (PH) is still debated and pre-procedural predictors of haemodynamic improvement after TEER in this setting are currently unknown. We investigated whether normalization of pulmonary artery wedge pressure (PAWP) in response to sodium nitroprusside (SNP) during baseline right heart catheterization might be predictive of a favourable haemodynamic response to MitraClip in patients with FMR and PH. Among 22 patients enrolled, 13 had a positive response to SNP (responders), nine were non-responders. At 6-months follow-up, responders showed a 33% reduction in PAWP and a 25% reduction in mean pulmonary artery pressure (PAP) (*P* = 0.002 and 0.004, respectively); no significant change occurred in non-responders. In patients with FMR and PH, pre-procedural vasodilator challenge with SNP may help define patients who may have haemodynamic improvement after TEER.

## Graphical Abstract

**Graphical Abstract:**
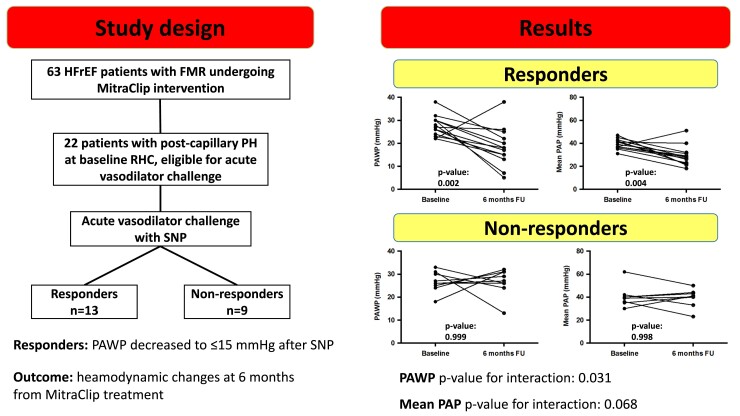

## Introduction

In patients with heart failure with reduced ejection fraction (HFrEF) and concomitant functional mitral regurgitation (FMR), pulmonary hypertension (PH) is a common finding^1^ associated with an increased risk of congestive HF and mortality.^2,3^ Data on transcatheter edge-to-edge repair (TEER) in patients with FMR and PH are scarce, with previous studies suggesting possible haemodynamic improvement after TEER in this subset of patients.^4–5^ However, potential predictors of haemodynamic improvement after TEER have not been investigated.

Sodium nitroprusside (SNP) can acutely improve cardiac filling pressures and reduce MR,^6^ mimicking the haemodynamic changes obtained with correction of MR and the consequent abolition of regurgitant volume. Accordingly, we aimed to assess whether normalization of pulmonary artery wedge pressure (PAWP) in response to a vasodilator challenge (AVC) with SNP during baseline right heart catheterization (RHC) might be indicative of a favourable haemodynamic response to MitraClip in patients with FMR and PH.

## Methods

All HFrEF patients affected by moderate to severe or severe (3+ or 4+/4+) FMR, who consecutively underwent MitraClip intervention between December 2012 and September 2019 at our Institution were enrolled in our prospective registry.

Right heart catheterization was performed in conscious patients before MitraClip procedure and at 6-months follow-up as an outpatient procedure. Study inclusion criteria were: (i) baseline post-capillary PH defined as mean pulmonary artery pressure (PAP) of >20 mmHg and PAWP >15 mmHg at RHC; (ii) AVC performed during pre-procedural RHC. Patients’ eligibility to AVC was defined according to heart transplant guidelines^7^ after baseline haemodynamic data were acquired, patients with PAP > 20 mmHg and PVR ≥ 3 Wood Units underwent AVC. The test was performed with up-titration of intravenous SNP: a starting dose of 10 μg/min was rapidly titrated until there was: (i) normalization in PAP; (ii) reduction in systolic blood pressure to <90 mmHg; or (iii) patient intolerance. Patients were then defined as responders when PAWP could be decreased to ≤15 mmHg. Pulmonary artery wedge pressure was measured at end-diastole and contributions of V waves were excluded by measurements.

Echocardiographic evaluation and grading of MR were assessed according to the 2013 European Association of Cardiovascular Imaging recommendations.^8^ The investigation conforms to the principles outlined in the Declaration of Helsinki, the protocol was approved by the Local Ethics Committee and for all patients an informed consent was acquired before each invasive haemodynamic evaluation.

Haemodynamic variables from baseline to 6-months follow-up were analyzed by fitting a mixed effect model for repeated measures (AVC response, time, and the interaction between AVC response × time were fixed, individual subjects as random-effects). The holm method was used for *post hoc* comparisons. Statistical significance was *P* < 0.05, analyses were performed in R environment.

## Results

### Patients

Of 63 consecutive HFrEF patients with FMR treated with MitraClip, 22 were eligible for inclusion in the study. The mean age was 64.7 (±9.9) years; 50% of patients were affected by post-ischaemic cardiomyopathy. At baseline, all patients were symptomatic despite guideline-directed medical therapy and in 77% of cases, at least one hospitalization for congestive HF within the previous year was reported.

### Baseline echocardiographic and right heart catheterization assessment

Baseline mean left ventricular (LV) ejection fraction was 26 (±4.7) %, LV end-diastolic volume indexed 140.5 (±35.3) mL/m^2^ and left atrial volume indexed 66.5 (±15.5) mL/m^2^ (*Table 1*).

**Table 1:** Baseline clinical and echocardiographic data and procedural results of the study population and their differences between responders and non-responders

	Overall	Responders	Non-responders	*P* value
	(*n* = 22)	(*n* = 13)	(*n* = 9)	
*Clinical characteristics*				
Age, years	64.7 ± 9.9	63 ± 12.3	67.2 ± 4.8	0.341
Male gender	16 (73)	9 (69)	7 (78)	0.658
BSA, m^2^	1.8 ± 0.2	1.7 ± 0.2	1.9 ± 0.2	0.249
Hypertension	10 (45.5)	5 (38.5)	5 (55.5)	0.429
Diabetes	4 (18)	2 (15.5)	2 (22)	0.683
Dyslipidaemia	13 (59)	6 (46)	7 (78)	0.138
eGFR, mL/min	58.2 ± 19.6	57 ± 22	60 ± 16.5	0.728
Atrial fibrillation	3 (13.5)	2 (15.5)	1 (11)	0.774
COPD	2 (9)	1 (7.5)	1 (11)	0.784
NYHA Classes III–IV	12 (54.5)	7 (54)	5 (55.5)	0.937
PH	22 (100)	13 (100)	9 (100)	
Ipc-PH	7 (32)	4 (31)	3 (33.5)	0.899
Cpc-PH	15 (68)	9 (69)	6 (66.5)	
Ischaemic cardiomyopathy	11 (50)	6 (46)	5 (55.5)	0.665
STS mortality, %	2.2 (1–4.7)	1.1 (1–3.7)	3.6 (1.3–4.7)	0.269
EuroSCORE II, %	4.9 (2.5–8)	4.5 (2.5–8)	5.4 (2.5–6.3)	0.867
COAPT-like^a^	11 (50)	5 (38.5)	6 (66.5)	0.193
*Past medical history*				
Previous AMI	11 (50)	6 (46)	5 (55.5)	0.665
Previous PCI	11 (50)	7 (54)	4 (44.5)	0.665
Previous CABG	5 (22.5)	2 (15.5)	3 (33.5)	0.323
Admission for HF in the last year	17 (77)	11 (84.5)	6 (66.5)	0.323
*GDMT at baseline*				
ACE-I/ARB	18 (82)	10 (77)	8 (89)	0.474
Beta-blocker	19 (86.5)	11 (84.5)	8 (89)	0.774
MRA	18 (82)	9 (69)	9 (100)	0.066
Furosemide	20 (91)	12 (92.5)	8 (89)	0.784
Furosemide, mg	58.5 ± 34.4	47.5 ± 29.5	74.5 ± 36	0.069
ICD	21 (95.5)	13 (100)	8 (89)	0.219
CRT	11 (50)	6 (46)	5 (55.5)	0.665
Echocardiographic features				
Mitral regurgitation				0.342
Moderate to severe (3+)	2 (9)	1 (7.5)	1 (11)	
Severe (4+)	20 (91)	12 (92.5)	8 (89)	
EROA, cm^2^	0.3 ± 0.1	0.3 ± 0.1	0.2 ± 0.09	0.251
RVol, mL	20 (15.5–26.5)	20 (17.5–27)	20 (15–26)	0.583
LVEF, %	26 ± 4.7	26.3 ± 5.2	25.5 ± 4.2	0.725
LVEDVi, mL/m^2^	140.5 ± 35.3	139.8 ± 41.4	141.6 ± 27.5	0.914
LVESVi, mL/m^2^	109.1 ± 29.9	107.3 ± 22.6	110.7 ± 36.6	0.823
LVEDD, mm	71.4 ± 7.9	69.8 ± 8.5	73.5 ± 6.8	0.292
LVESD, mm	63.6 ± 8.8	62.2 ± 8.9	65.5 ± 8.8	0.397
LAVi, mL/m^2^	66.5 ± 15.5	72.5 ± 14.4	60.4 ± 14.7	0.098
PASP, mmHg	49.3 ± 12.7	48.5 ± 11.2	50.3 ± 15.3	0.753
PASP ≥ 50 mmHg	10 (45.5)	7 (54)	3 (33.5)	0.342
TAPSE, mm	17.1 ± 3	17.5 ± 3.1	16.5 ± 3	0.505
TAPSE/PASP	0.3 ± 0.08	0.31 ± 0.08	0.28 ± 0.08	0.409
TR > 2	13 (59)	5 (38.5)	8 (89)	0.027
*Procedural data*				
Procedural success	20 (91)	12 (92.5)	8 (89)	0.784
*N* of clips implanted	1.7 ± 0.5	1.8 ± 0.5	1.5 ± 0.5	0.232
Residual MR	1.5 ± 0.5	1.6 ± 0.5	1.4 ± 0.5	0.452
Post-clip MV gradient	2.9 ± 1.4	3.2 ± 1.6	2.6 ± 1.1	0.385

BSA, body surface area; eGFR, estimated glomerular filtration rate; COPD, chronic obstructive pulmonary disease; PH, pulmonary hypertension; Cpc-PH, combined post- and pre-capillary pulmonary hypertension; Ipc-PH, isolated post-capillary pulmonary hypertension; AMI, acute myocardial infarction; PCI, percutaneous coronary intervention; CABG, coronary artery bypass graft; HF, heart failure; ACE-I, angiotensin-converting enzyme inhibitor; ARB, angiotensin receptor blocker; ICD, implantable cardioverter defibrillator; CRT, cardiac resynchronization therapy; EROA, effective regurgitant orifice area; RVol, regurgitant volume; LVEF, left ventricle ejection fraction; LVESDi, left ventricular end-systolic diameter indexed; LVESVi, left ventricular end-systolic volume indexed; LVEDD, left ventricular end-diastolic diameter; LVESD, left ventricular end-systolic diameter; LAVi, left atrial volume index; PASP, pulmonary arterial systolic pressure; TAPSE, tricuspid annular plane systolic excursion; TR, tricuspid regurgitation; MR, mitral regurgitation; MV, mitral valve.

a Patients fulfilling the COAPT inclusion criteria (PASP < 70 mmHg, LVESD < 70 mm, LVEF 20–50%, absence of moderate to severe right ventricular dysfunction, absence of severe tricuspid regurgitation).

At baseline RHC, all patients presented post-capillary PH (mean PAP 39.5 25th–75th percentile: 36–42 mmHg, PAWP 27.2 ± 4.4 mmHg), with combined pre-capillary and post-capillary PH in 15 cases (68%). The AVC was adequately performed in all cases: 13 patients had a positive response (responders), while nine were non-responders.

Comparing clinical, echocardiographic, and haemodynamic characteristics between the two study groups (responders vs. non-responders), no statistical differences were found except for tricuspid regurgitation, which was greater in non-responders (*Table 1*).

Characteristics of patients who underwent MitraClip treatment during the same time period but for whom study eligibility criteria were not satisfied are reported in Supplementary material online, *Table S1*.

### Procedural and follow-up results

Procedural success (according to Mitral Valve Academic Research Consortium criteria^9^) was achieved in 20 patients (91%), with no difference between responders and non-responders.

During the follow-up period, no significant modification in HF drug dosages occurred.

At 6-months, RHC showed a significant increase in cardiac index (+0.45, 95% CI: +0.61 to +0.29 L/min/m^2^, *P* < 0.001) and a significant drop of PAWP (−5.4, 95% CI: −0.8 to –10.1 mmHg, *P* = 0.023), with consensual reduction of mean PAP (−6.5, 95% CI: −1.9 to –10.9 mmHg, *P* = 0.012).

**Figure 1:**
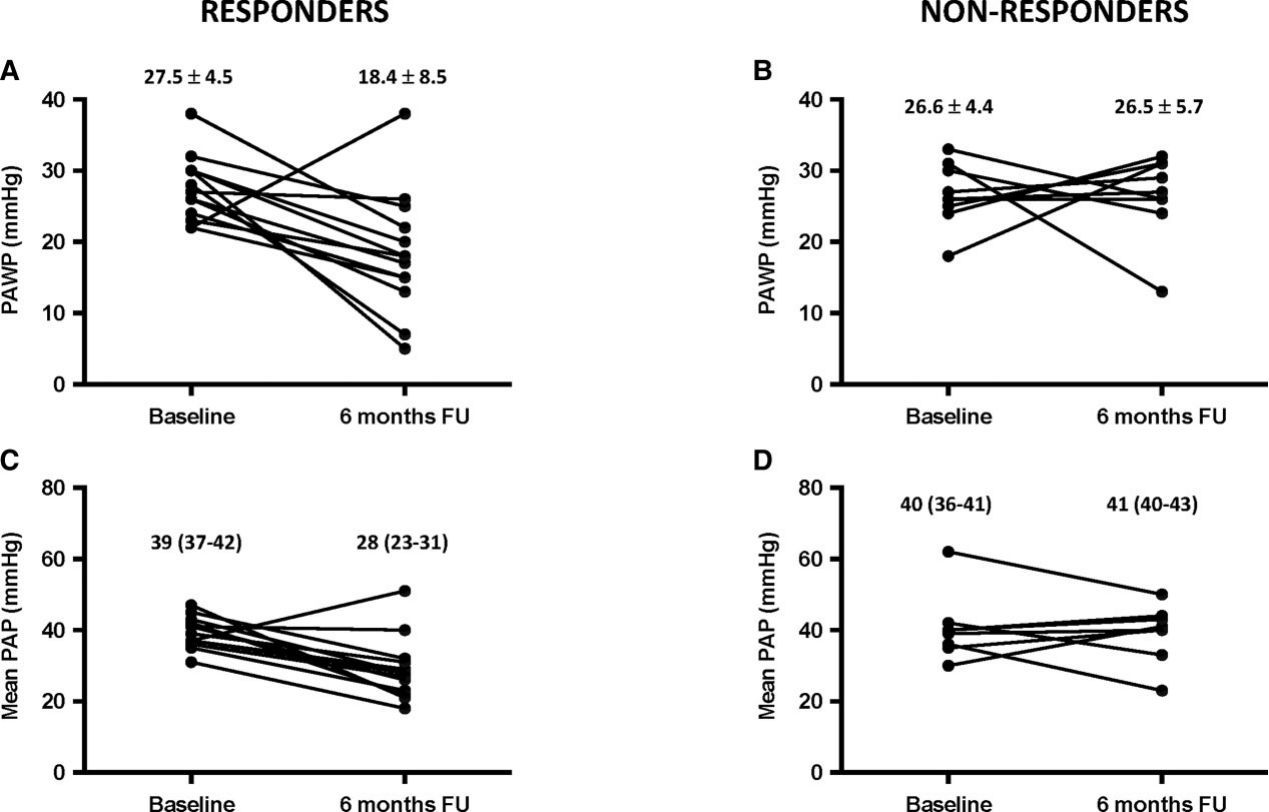
Pulmonary artery wedge pressure and mean pulmonary artery pressure before and 6 months after MitraClip procedure in patients with a positive (responders) and negative (non-responders) response to acute vasodilator challenge.

Comparing haemodynamic changes between the two groups, despite a similar improvement in cardiac index was observed, responders showed a 33% reduction in PAWP (*P* = 0.002) while no change occurred in non-responders (*P* for interaction = 0.031). Similarly, a 25% reduction in mean PAP (*P* = 0.004) was observed in responders, with no change in non-responders (*P* for interaction = 0.068), *Table 2* and *Figure 1*.

**Table 2:** Changes in haemodynamic parameters six months after MitraClip intervention among responders and non-responders

	Baseline		6-months follow-up		Time effect	Group effect	Time × group effect
	Responders	Non-responders	Responders	Non-responders	*P*-value	*P*-value	*P*-value
	(*n* = 13)	(*n* = 9)	(*n* = 13)	(*n* = 9)			
*Haemodynamic parameters*							
Cardiac index, L/min/m^2^	1.6 (1.4–1.7)	1.7 (1.3–1.9)	2.2 (1.8–2.4)	2.2 (1.8–2.5)	<0.001	0.610	0.911
Systolic PAP, mmHg	57 (48–65)	54 (53–65)	45 (40–46.5)	60 (57–71)	<0.001	0.010	0.056
Mean PAP, mmHg	39 (37–42)	40 (36–41)	28 (23–31)	41 (40–43)	<0.001	0.006	0.068
Diastolic PAP, mmHg	27.1 ± 4.9	26.3 ± 6.5	17.8 ± 7.2	26.3 ± 5.3	0.011	<0.001	0.034
PAWP, mmHg	27.5 ± 4.5	26.6 ± 4.4	18.4 ± 8.5	26.5 ± 5.8	<0.001	0.003	0.031
RAP, mmHg	8.7 ± 3.3	8.5 ± 1.9	5.6 ± 3.4	10.2 ± 5.8	0.010	0.033	0.066
PVR, WU	3.9 ± 1.8	4.3 ± 2.4	3.0 ± 1.5	3.6 ± 1.5	0.046	0.726	0.405
PCA, mL/mmHg	1.6 ± 0.3	1.4 ± 0.3	2.2 ± 0.3	1.4 ± 0.5	0.031	0.110	0.417
TAPSE/PASP, mmHg	0.31 ± 0.08	0.28 ± 0.08	0.42 ± 0.14	0.28 ± 0.11	0.035	<0.001	0.031

CI, cardiac index; PAP, pulmonary artery pressure; PAWP, pulmonary artery wedge capillary; RAP, right atrial pressure; PVR, pulmonary vascular resistance; PCA, pulmonary compliance artery; TAPSE, tricuspid annular plane excursion; PASP, pulmonary artery systolic pressure.

## Discussion

Our study on patients with HFrEF, FMR, and PH showed that PAWP normalization following AVC with SNP during pre-procedural RHC was a good predictor of haemodynamic improvement after MitraClip treatment.

Data on positive haemodynamic changes after MitraClip procedure in patients with MR and PH have been previously reported.^4,5^ Recently, a *post hoc* analysis of the COAPT trial confirmed these results in patients with HFrEF and FMR,^3^ a clinical setting where PH is a common finding often undertreated, negatively affecting prognosis.^2,3^ In each of these studies,^3,4^ failure to improve vascular pulmonary haemodynamics after Mitraclip procedure was reported as an independent predictor of worse outcomes.

However, pre-procedural predictors of haemodynamic improvement after TEER in these patients are currently unknown.

In the present series of patients with FMR and PH undergoing pre-procedural AVC with SNP, right heart haemodynamics after MitraClip improved significantly more in SNP responders than in non-responders. Sodium nitroprusside is a potent vasodilator that can acutely reduce LV afterload, improve cardiac filling pressures, reduce MR, and increase cardiac output in patients with HFrEF.^6^ The decrease of the trans-mitral regurgitant volume after a successful MitraClip similarly leads to a reduction of left atrial overload with an increase in cardiac forward output.^10^ The substantial improvement in PAWP and mean PAP after MitraClip in SNP responders, but not in SNP non-responders, allows us to hypothesize that the normalization in PAWP after SNP infusion may be indicative of a relevant role of MR in the pathophysiology of the individual patient. As a consequence, in this context, the correction of MR with TEER could lead to a reduction in left atrial and pulmonary arterial pressures. Therefore, our data suggest that pre-procedural AVC may help in defining MitraClip patient selection.

These considerations remain purely speculative and hypothesis-generating because the study is limited by a small sample size of selected advanced HF patients from a single centre. Therefore, the possible role of AVC in the context of TEER patient selection has to be confirmed in larger prospective studies. Nevertheless, these data add a new perspective in the decisional pathway of patient selection for TEER treatment, particularly for those patients with advanced HF and PH that would be formally ineligible for TEER by current selection criteria.

## Supplementary Material

zuac053_Supplementary_Data

## Data Availability

The data underlying this article will be shared on reasonable request to the corresponding author.
