# Supplementary material for: Response to acute vasodilator challenge and haemodynamic modifications after MitraClip in patients with functional mitral regurgitation and pulmonary hypertension
Source: Eur Heart J Acute Cardiovasc Care. 2022 May 7;11(6):464–9. doi: 10.1093/ehjacc/zuac053 (PMC9459870; doi:10.1093/ehjacc/zuac053)
Supplement: zuac053_Supplementary_Data [file zuac053_supplementary_data.docx]

|  | Study Population  (n = 22) | Not-enrolled Population  (n = 41) | p value |
| --- | --- | --- | --- |
| Clinical Characteristics |  |  |  |
| Age, years | 64.7±9.9 | 68.2±8.8 | 0.158 |
| Male gender | 16 (73) | 33 (80.5) | 0.480 |
| BSA, m2 | 1.8±0.2 | 1.8±0.17 | 0.778 |
| Hypertension | 10 (45.5) | 26 (63.5) | 0.170 |
| Diabetes | 4 (18) | 10 (24.5) | 0.572 |
| Dysplipidemia | 13 (59) | 27 (69) | 0.423 |
| eGFR, mL/min | 58.2±19.6 | 57.5±21.3 | 0.893 |
| Atrial Fibrillation | 3 (13.5) | 8 (20) | 0.530 |
| COPD | 2 (9) | 6 (14.5) | 0.529 |
| NYHA class III-IV | 12 (54.5) | 25 (61) | 0.621 |
| PH | 22 (100) | 33 (82) | 0.035 |
| Ischemic cardiomyopathy | 11 (50) | 26 (63.5) | 0.303 |
| STS mortality, % | 2.2 (1-4.7) | 2.3 (1-5.4) | 0.562 |
| EuroSCORE II, % | 4.9 (2.5-8) | 4.7 (2.2-7) | 0.616 |
| COAPT-like^a^ | 11 (50) | 21 (51) | 0.926 |
| Past medical history |  |  |  |
| Previous AMI | 11 (50) | 26 (65) | 0.249 |
| Previous PCI | 11 (50) | 24 (58.5) | 0.516 |
| Previous CABG | 5 (22.5) | 7 (17) | 0.586 |
| Admission for HF in the last year | 17 (77) | 34 (83) | 0.586 |
| GDMT at baseline |  |  |  |
| ACE-I/ARB | 18 (82) | 34 (83) | 0.912 |
| Beta-blocker | 19 (86.5) | 33 (80.5) | 0.558 |
| MRA | 18 (82) | 25 (61) | 0.090 |
| Furosemide | 20 (91) | 37 (90) | 0.932 |
| Furosemide, mg | 58.5±34.4 | 56.3±38.3 | 0.824 |
| ICD | 21 (95.5) | 27 (66) | 0.009 |
| CRT | 11 (50) | 12 (29) | 0.103 |
| Echocardiographic Features |  |  |  |
| Mitral Regurgitation |  |  | 0.031 |
| Moderate to severe (3+) | 2 (9) | 15 (36.5) |  |
| Severe (4+) | 20 (91) | 26 (63.5) |  |
| EROA, cm2 | 0.3±0.1 | 0.3±0.1 | 0.724 |
| RVol, mL | 20 (15.5-26.5) | 23 (15-42) | 0.344 |
| LVEF, % | 26±4.7 | 29.5±9 | 0.098 |
| LVEDVi, mL/m^2^ | 140.5±35.3 | 147.5±51.8 | 0.600 |
| LVESVi, mL/m^2^ | 109.1±29.9 | 108.5±48.5 | 0.961 |
| LVEDD, mm | 71.4±7.9 | 68±9 | 0.162 |
| LVESD, mm | 63.6±8.8 | 58.8±11.6 | 0.123 |
| LAVi, mL/m^2 §^ | 66.5±15.5 | 67.9±17.2 | 0.769 |
| PASP, mmHg | 49.3±12.7 | 44.4±15 | 0.215 |
| PASP ≥ 50 mmHg | 10 (45.5) | 15 (37) | 0.533 |
| TAPSE, mm | 17.1±3 | 18.2±3 | 0.196 |
| TR> 2 | 13 (59) | 11 (26.5) | 0.019 |
| Haemodinamyc parameters |  |  |  |
| Cardiac index, L/min/m^2^ | 1.65 (1.4-1.9) | 1.94 (1.6-2.14) | 0.027 |
| Systolic PAP, mmHg | 57 (51-65) | 48 (34-63) | 0.015 |
| Mean PAP, mmHg | 39.5 (36-42) | 29 (21-38) | <0.001 |
| Diastolic PAP, mmHg | 26.7±5.5 | 19.2±7.8 | <0.001 |
| PAWP, mmHg | 27.2±4.4 | 20.9±8.5 | 0.002 |
| RAP, mmHg | 8.6±2.7 | 6.2±3.9 | 0.013 |
| PVR, WU | 4.0±2 | 2.2±0.9 | 0.002 |
| PCA, ml/mmHg | 1.5±0.7 | 2.0±0.9 | 0.028 |
| TAPSE/PASP, mmHg | 0.3±0.08 | 0.4±0.2 | 0.006 |
| Procedural data |  |  |  |
| Procedural success | 20 (91) | 35 (85.5) | 0.529 |
| N of clips implanted | 1.7±0.5 | 1.9±0.5 | 0.307 |
| Residual MR | 1.5±0.5 | 1.5±0.7 | 0.985 |
| Post-clip MV gradient | 2.9±1.4 | 2.9±1 | 0.977 |

Legend: BSA: body surface area; eGFR: estimated glomerular filtration rate; COPD: chronic obstructive pulmonary disease; PH: pulmonary hypertension; Cpc-PH: combined post- and pre-capillary pulmonary hypertension; Ipc-PH: isolated post-capillary pulmonary hypertension; AMI: acute myocardial infarction; PCI: percutaneous coronary intervention; CABG: coronary artery bypass graft; HF: heart failure; ACE-I: angiotensin-converting enzyme inhibitor; ARB: angiotensin receptor blocker; ICD: implantable cardioverter defibrillator; CRT: cardiac resynchronization therapy; EROA: effective regurgitant orifice area; RVol: regurgitant volume; LVEF: left ventricle ejection fraction; LVESDi: left ventricular end-systolic diameter indexed; LVESVi: left ventricular end-systolic volume indexed; LVEDD: left ventricular end-diastolic diameter; LVESD: left ventricular end-systolic diameter; LAVi: left atrial volume index; PASP: pulmonary arterial systolic pressure; TAPSE: tricuspid annular plane systolic excursion; TR: tricuspid regurgitation. CI: cardiac index; PAP: pulmonary artery pressure; PAWP: pulmonary artery wedge capillary; RAP: right atrial pressure; PVR: pulmonary vascular resistance; PCA: pulmonary compliance artery; MR: mitral regurgitation; MV: mitral valve.

a. Patients fulfilling the COAPT inclusion criteria (PASP < 70 mmHg, LVESD < 70 mm, LVEF 20-50%, absence of moderate to severe right ventricular dysfunction, absence of severe tricuspid regurgitation).
